# Transcriptome assembly and expression profiling of the molecular responses to cadmium toxicity in cerebral ganglia of wolf spider *Pardosa pseudoannulata* (Araneae: Lycosidae)

**DOI:** 10.1007/s10646-017-1885-1

**Published:** 2018-01-03

**Authors:** Huilin Yang, Yuande Peng, Yixue Shi, Jianxiang Tian, Juan Wang, Xianjin Peng, Chunliang Xie, Xiang Xu, Qisheng Song, Zhi Wang, Zhiyue Lv

**Affiliations:** 1 College of Orient Science & Technology, Hunan Agriculture University, No. 1 Nongda Road, Changsha, 410128 Hunan China; 2 College of Bioscience and Biotechnology, Hunan Agriculture University, No. 1 Nongda Road, Changsha, 410128 Hunan China; 3 Institute of Bast Fiber Crops, Chinese Academy of Agricultural Sciences, Changsha, 410205 Hunan China; 4 College of Continuing Education, Hunan Agriculture University, No. 1 Nongda Road, Changsha, 410128 Hunan China; 5 College of Life Science, Hunan Normal University, Changsha, 410006 Hunan China; 6 Division of Plant Sciences, University of Missouri, Columbia, MO 65211 USA; 7 Department of Parasitology, Zhongshan School of Medicine, Sun Yat-Sen University, Guangzhou, 510080 China

**Keywords:** Cadmium, Transcriptomic, *P. pseudoannulata*, Cerebral ganglion

## Abstract

Cadmium (Cd) is a heavy metal that can cause irreversible toxicity to animals, and is an environmental pollutant in farmlands. Spiders are considered to be an excellent model for investigating the impacts of heavy metals on the environment. To date, the changes at the molecular level in the cerebral ganglia of spiders are poorly understood. Cd exposure leads to strong damage in the nervous system, such as apoptosis and necrosis of nerve cells, therefore we conducted a transcriptomic analysis of *Pardosa pseudoannulata* cerebral ganglia under Cd stress to profile differential gene expression (DGE). We obtained a total of 123,328 assembled unigenes, and 1441 Cd stress-associated DEGs between the Cd-treated and control groups. Expression profile analysis demonstrated that many genes involved in calcium signaling, cGMP—PKG signaling, tyrosine metabolism, phototransduction–fly, melanogenesis and isoquinoline alkaloid biosynthesis were up-regulated under Cd stress, whereas oxidative phosphorylation-related, nervous disease-associated, non-alcoholic fatty liver disease-associated, and ribosomal-associated genes were down-regulated. Here, we provide a comprehensive set of DEGs influenced by Cd stress, and heavy metal stress, and provide new information for elucidating the neurotoxic mechanisms of Cd stress in spiders.

## Introduction

Cadmium (Cd) is a non-essential heavy metal that is a non-biodegradable pollutant across the world (Mehinto et al. 2014; Nemmiche et al. 2011). Cd is highly neurotoxic in animals, and damages induced by high levels of Cd include changes in brain morphology, disruption of the brain barrier of the central nervous system, changes in neurotransmitter content and enzyme activity, and alterations in brain metabolism (Murthy et al. 1989). Interestingly, Cd toxicity can have different effects within individuals of the same species. For example, Cd distribution in the ganglia is different in various strains of mice, resulting in differing effects of Cd to the trigeminal ganglia (Habeebu et al. 2001).

Spiders are considered to be an excellent invertebrate model to investigate Cd contamination, based on species diversity, geographic distribution and large appetite (Jung et al. 2005; Yang et al. 2016; Li et al. 2016). Previous studies have demonstrated that Cd can accumulate in spiders and affect their physiological and ecological traits (Wilczek et al. 2008; Jung and Lee 2012; Jöst and Zauke 2008; Eraly et al. 2010). More than 5000 farmland spider species belonging to 3000 genera have been described in the world, and these spiders are often the most generalist predator in farmlands (Samu and Szinetár 2002). *Pardosa pseudoannulata* belongs to Lycosidae, and is one of the most common wandering spiders in fields across the rice growing regions of China (Li et al. 2001). Li et al. (2016) carried out a whole body transcriptome analysis of the spider *P. pseudoannulata* exposed to Cd, and identified a total of 2939 differentially expressed genes (DEGs), including multiple candidate genes involved in defense and detoxification of Cd, as well as genes encoding glutathione metabolism related enzymes and heat shock proteins.

The rapid development of high-throughput DNA sequencing technology has facilitated detailed and comprehensive analysis of the transcriptomes and genomes of non-model organisms (Margulies et al. 2005; Wicker et al. 2006; Liu et al. 2013; Wang et al. 2016a, 2017). Meng et al. (2013) found over 3000 differentially expressed genes (DEGs) in the digestive glands of Japanese scallops *Mizuhopecten yessoensis* upon Cd exposure, of which 154 DEGs were involved in ABC transporters, glycine, serine and threonine metabolism, steroid hormone biosynthesis and glutathione metabolism. Sun et al. (2016) conducted transcriptomic analysis of Cd-treated freshwater crab hepatopancreas tissues, and reported increases in expression of genes involved in macromolecular metabolism, oxidative phosphorylation, detoxification and anti-oxidant defense. In the hepatopancreas of the razor clam *Sinonovacula constricta* exposed to Cd, genes showing significant expression level changes included those involved in metabolic processes, cellular processes, and ROS production-related genes, such as heat shock proteins 32, metallothionein, and glutathione (Wang et al. 2016b).

Here, we conducted a comparative analysis of DGEs in the nervous system of *P. pseudoannulata* in response to Cd stress to identify associated biological processes and pathways, and to reveal the type of processes that may be disrupted in the cerebral ganglion of spider. To our knowledge, this is the first report on the transcriptome profiling of the cerebral ganglion of *P. pseudoannulata*, thus improving our understanding of the mechanism by which heavy metal neurotoxins impact the spider.

## Materials and methods

### Animal materials and treatments

*P. pseudoannulata* specimens were collected from experimental farmland in the Hunan Academy of Agricultural Science, Changsha (27^°^51′N, 111°53′E), Hunan Province, China. Spiders were placed individually in cylindrical glass tubes with a moist cotton ball (12 × 100 mm). Spiders were fed daily with *Drosophila melanogaster* that were reared on cadmium chloride medium (1.0 mg/L) or non-Cd medium. All tubes were marked and maintained in an artificial climate chamber maintained at 30 °C, 70% RH and L:D 10:14 photoperiod.

A total of 120 spiders were used in the experiment. To determine Cd concentrations, there was a control group and an experimental group each with three replicates with each replicate containing 20 spiders. Spiders were observed twice a day at 9 a.m. and 9 p.m. No mortality was observed during the entire experimental period. The spiders were dissected within an ice bath after 30 days of treatment, the cerebral ganglion in the Cd-treated and control spiders were collected, immediately frozen in liquid nitrogen and stored at −70 °C for RNA extraction.

### Spider sample digestion and Cd content determination

Each sample weighed between 0.2–0.5 g and the samples were washed with 1% nitric acid three times, then placed into a tube containing 1.5 mL hydrochloric acid and 4.5 mL nitric acid for digestion. The samples were digested by a digestion instrument (SPH620, Alva instrument) at 90 °C for 1 h and 120 °C for 2 h, and then the cadmium content of the samples were determined by ICP (ICPE-9000) at 228.3 nm.

### RNA extraction, transcriptome sequencing and *de novo* assembly

RNA extraction and transcriptome sequencing were conducted by Oebiotech Enterprise, Shanghai. RNA was extracted from a pooled samples of 40 cerebral ganglions dissected from the experimental or control spiders. The quality of RNAs were determined with a NanoDrop ND-1000 spectrophotometer (NanoDrop Technologies Inc., Rockland, DE, USA), and only samples with 1.8 ≤ OD260/OD280 ≤ 2.1 were used for generating the transcriptome. RNA sequencing libraries were constructed and sequenced on an Illumina Hiseq^TM^ 2500.

Raw sequences were obtained by removing adapter sequences and low quality sequences by TGICL (Pertea et al. 2003). The remaining clean reads were used for *de novo* assembly by Trinity (Grabherr et al. 2011). Unique unigenes were generated by removing redundant sequences, and these unigenes were used for downstream bioinformatics analysis.

### Functional annotation

All unigenes were compared to protein databases, including the NCBI non-redundant protein (Nr) database and Swiss-prot database using Blastx. The Blast2GO program and WEGO software were used to obtain GO (Gene ontology) annotation for all unigenes (Conesa et al. 2005; Ye et al. 2006). COG (clusters of orthologous group) classification and KEGG (Kyoto encyclopedia of genes and genomes database) metabolic pathway annotation of unigenes were assigned by Blastx searching against KEGG and COG databases (Tatusov et al. 2003). The best aligned results were used to determine potential function of the unigenes. The parameter E-value < 1e−5 of BLASTx was taken as a threshold of significant similarity.

### Identification of differentially expressed genes (DEGs) and functional annotation

The FPKM (fragments per kb per million reads) method was used to quantify gene expression levels (Trapnell et al. 2010). The DEGseq software package was used to screen DEGs using the negative binomial distribution algorithm (Wang et al. 2010). False discovery rate (FDR) < 0.01 was used as the threshold to determine significant differences in gene expression. GO and KEGG pathway analyses were conducted for all DEGs. The hypergeometric test was used to identify significantly enriched GO terms in DEGs. The calculated *p* values were then corrected using the Bonferroni Correction, with corrected *p* value ≤ 0.001 as a threshold. GO terms fulfilling this condition were defined as significantly enriched GO terms in DEGs.

Similarly, pathway enrichment analysis was conducted to identify significantly enriched pathways including metabolic pathways or signal transduction pathways in DEGs, using a *p* value of ≤0.05 as a threshold.

### Quantitative real-time PCR analysis

Quantitative real-time PCR (qPCR) was used to verify the transcriptome results. Total RNA was extracted from each sample with TRIzol (Invitrogen, USA) and subjected to DNase I treatment (Promega, USA) according to the manufacturers’ protocols. cDNA was synthesized with a RevertAid™ H Minus First Strand cDNA Synthesis Kit (Fermentas Lithuania) and qPCR was performed using the ABI 7900 HT system (ABI, USA). The experiment was repeated three times, and expression levels of each gene were normalized to 18S ribosomal RNA and calculated using the 2^-ΔΔCt^ method. All primers were designed using the using the Primer 3.0 program (Table 1).

**Table 1:** List of primers used for qPCR

No.	Gene symbol	Forward primer	Reverse primer
1	CL9578Contig1	GAATTTCGACGAGATAGACCG	TTATTCTTTCCTGCCACGC
2	CL5353Contig1	CACACTGTCTACAAAGTCCTG	GAACCACGAATTGGGAGAT
3	CL8937Contig1	CCAACGCAGACAATCAGAAGA	ACGACAGGATCAGGTAGG
4	CL911Contig1	GTCTGCCTGTCTTTCCTTG	TTCAGCAGTAACACTCTCGTAG
5	CL14434Contig1	CAGCTTCGACGAGTTCAG	ATGTAAGTCTCGATCACGTTG
6	comp228568_c0_seq3	CTGCTGCTATGTAAGGATGTCA	ACTCTTGGCTGCTTTGGAA
7	CL17095Contig1	GCTACAACGATCTCTTAGCCT	TAGCCGTCCAGATAGTTTGC
8	CL1Contig1327	GCTCCTTCGCTGTTTAGTC	GCAGAGAAAGTTGTTGGCA
9	comp42711_c0_seq2	AGTGCATTCAAGTGAAGGT	ATCATCAACACATTTGAACAGC
10	CL21551Contig1	CCGAACAGGCTCAAGAAG	TCACGGAACCTCCGTAGATA
11	18S	CGGCTACCACATCCAAGGAA	GCTGGAATTACCGCGGCT

qPCR data were analyzed using a *t*-test with SPSS 17.0 software. Significant differences at *p* < 0.05 were designated with *, and data were presented as the mean ± SE.

## Results

### Amount of cadmium (Cd) in *P. pseudoannulata*

To test the effect of Cd on *P. pseudoannulata*, we fed spiders with fruit flies that were reared on standard media (control), or Cd-containing media (treatment) continuously for 30 days. Control spiders did not contain any detectable Cd. Cd treated spiders had increasing Cd levels with feeding time, reaching 7.27 µg/g Cd by day 30.

### *De novo* assembly of unigenes

We generated transcriptomes of *P. pseudoannulata* cerebral ganglion of treated and control spiders and obtained 47,661,402 clean DNA sequences reads with 5,957,675,250 bases after removing the low-quality reads, with Q30 percentages of 92.10%. De novo assembly of the clean reads was performed and 123,328 unigenes with an average length of 1040.73 bp were obtained. The N50_s_ of the unigenes were 1554, and 36,991 unigenes were over 1 kb (Table 2).

**Table 2:** Summary statistics of de novo transcriptome assembly

	All ≥200 bp	≥500 bp	≥1000 bp	N50	Total length	Max length	Min length	Average length
Unigene	123,328	76,190	36,991	1554	128,350,740	23,679	301	1040.73

### Functional annotation

**Fig. 1:**
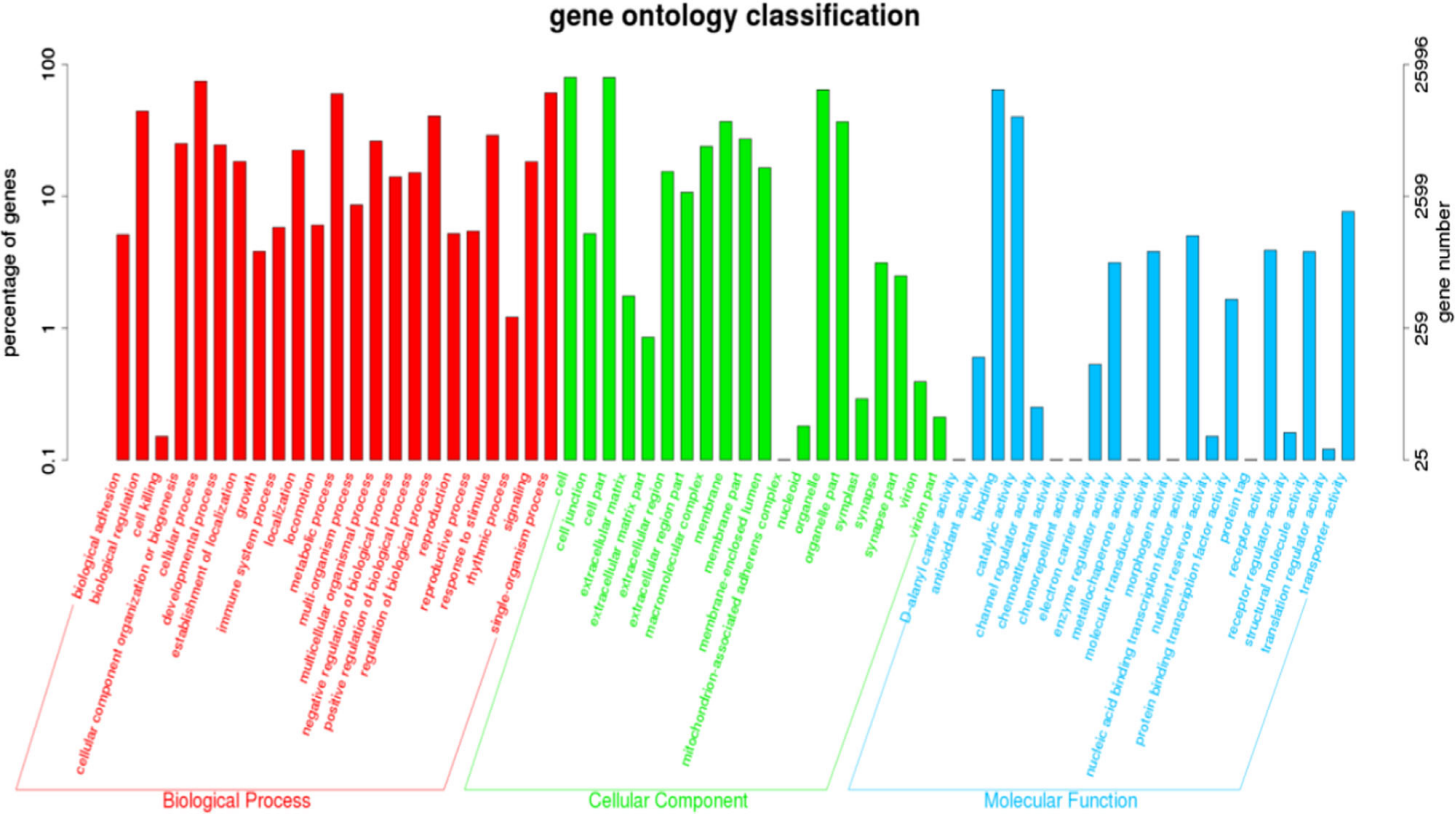
GO classification of all unigenes identified in the transcriptome of the cerebral ganglia of *P. pseudoannulata*. Analysis was conducted using Blast2Go (level 2), and red bars represent GO processes under biological processes, green under cellular component, and blue under molecular function

Among all unigenes, 39,029 (31.65% of all unigenes) matched to the Nr database, 28,049 (22.74%) to the Swiss-prot database, 25,031 (20.30%) to COG, 25,996 (21.08%) to the GO database, and 10,254 (8.31%) to the KEGG database (Table 3). From the results of annotation of the Nr database, 454 unigenes (1.16% of the total) showed similar sequences with the genome of *Zootermopsis nevadensis*, 442 unigenes (1.13% of the total) with the genome of *Acyrthosiphon pisum*, 358 unigenes (0.92% of the total) with the genome of *Ixodes scapularis*. A total of 28,113 unigenes (72.04%) did not match those in any species. GO annotation enriched unigenes were divided into three clusters: biological process (BP), cellular component (CC) and molecular function (MF). Biological processes made up the majority of the annotated unigenes (66.71%), followed by molecular functions (22.79%) and cell components (10.49%). The major subcategories were “cell” and “cell part” in cell components, and “cellular process” within biological processes (Fig. 1).

**Table 3:** Functional annotation of cerebral ganglion of *P. pseudoannulata* transcriptome

Database	NR	SWISSPROT	KOG	KEGG	GO
Annotation_numbers	39,029	28,049	25,031	10,254	25,996
Annotation_ratio	31.65%	22.74%	20.30%	8.31%	21.08%

**Fig. 2:**
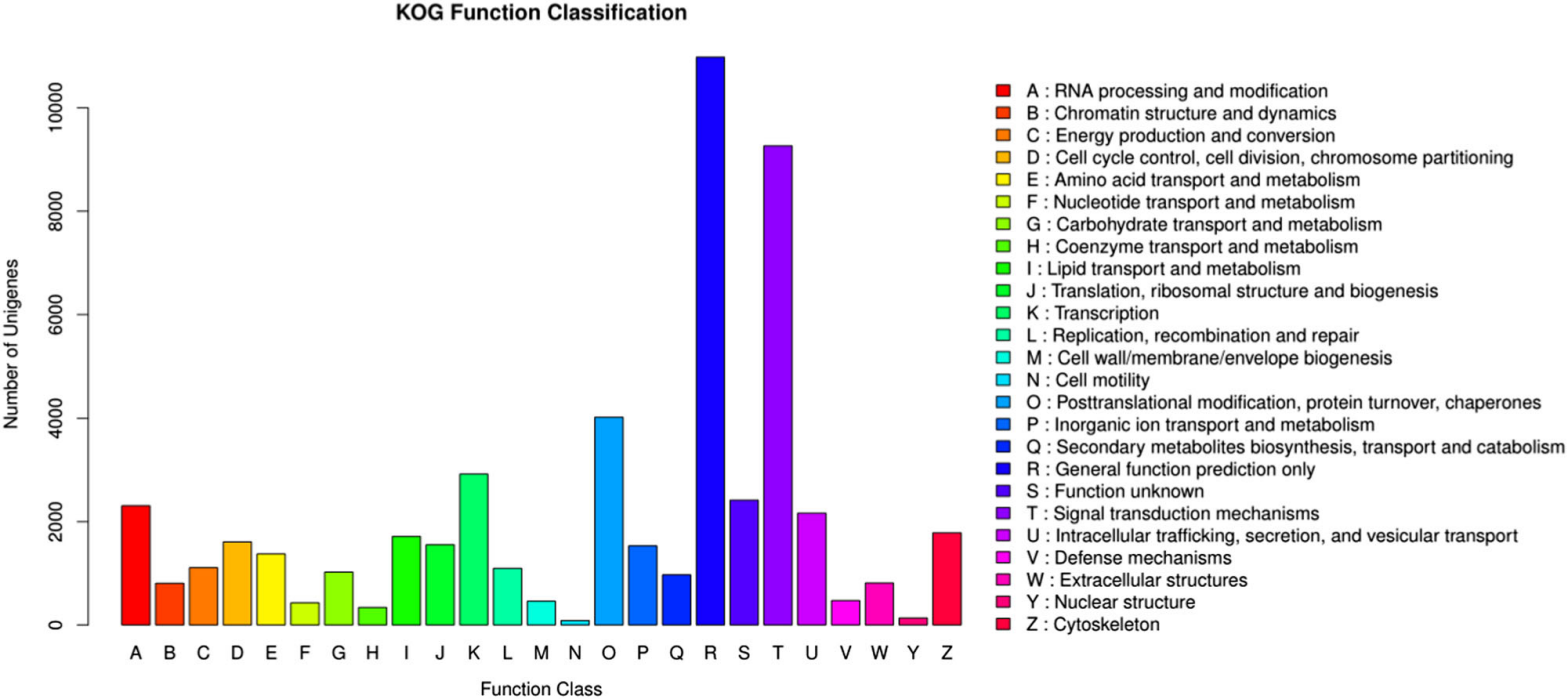
COG classification of unigenes in the cerebral ganglion transcriptome of *P. pseudoannulata*

A total of 25,031 unigenes had significant matches in the COG database. Among these, the “General function prediction only” cluster (21.37%) was the largest, followed by “Signal transduction mechanisms” (18.04%), “Post-translation modification, protein turnover, chaperones”, and “transcription” (7.83%) (Fig. 2). In addition, 10,254 unigenes were categorized into 333 KEGG pathways. Five largest categories were pathways in cancer (human diseases/cancers), ribosome (genetic information processing/translation), Huntington’s disease (human diseases/neurodegenerative diseases), protein processing in endoplasmic reticulum (genetic information processing/folding, sorting and degradation), and lysosome (cellular processes/transport and catabolism).

### Differential gene expression

**Fig. 3:**
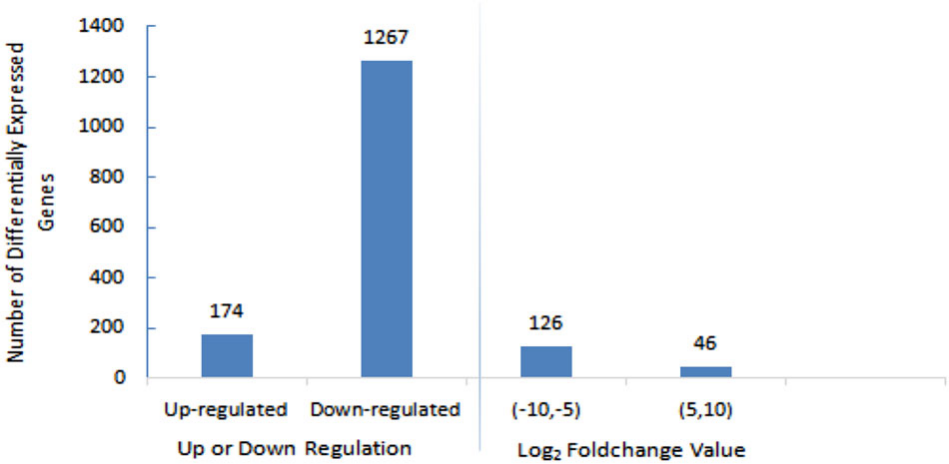
Distribution of differently expressed unigenes between cerebral ganglia of Cd-treated and control *P. pseudoannulata*

A total of 1441 DEGs were identified, of which 174 were significantly up-regulated and 1267 were significantly down-regulated (*p* ≤ 0.01). Among all DEGs, 126 had fold changes ranging from −5 to −10 and the remaining 46 had fold changes ranging from 5 to 10 (Fig. 3). These results show that Cd treatment altered gene expression in the cerebral ganglion of Cd-treated spiders.

### Functional enrichment analysis of DEGs

To explore the expression of DEGs in the ganglia of spiders in the present study, we conducted GO term functional annotation based on GO classification and pathway enrichment analysis. We found that 48 GO terms (FDR < 0.001) were significantly enriched. Among the GO term functional annotation, biological process (BP) (26 GO terms, 102 genes), cellular component (CC) (8 GO terms, 304 genes) and molecular function (MF) (14 GO terms, 141 genes) accounted for 54.16, 16.67 and 29.17% respectively (Figs. 1–3). The main enriched subcategories in biological process were “proteolysis involved in cellular protein catabolic process”, “cellular iron ion homeostasis” and “phototransduction”. The two main categories within the molecular function category were “structural constituent of cuticle” and “chitin binding”, accounting for 41.14% of the DEGs in this category. In the cellular component category, “extracellular region” and “endoplasmic reticulum membrane” were the main enriched subcategories (51.46%).

**Fig. 4:**
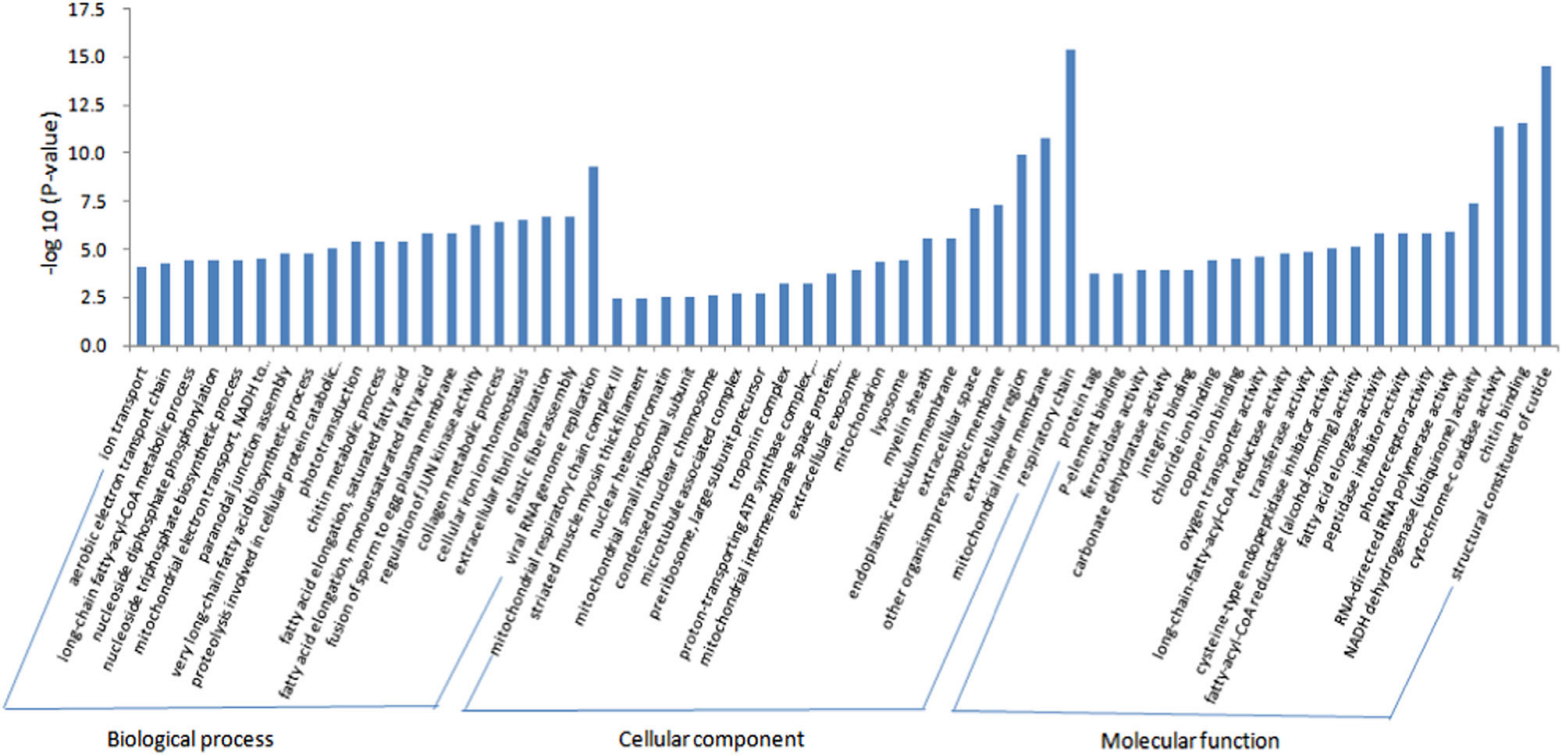
GO enrichment analysis of top 30 terms represented in the DEGs. The abscissa is the Unigene number, and the vertical axis lists the GO term

Top GOs were further used to identify enriched GO terms (Fig. 4). The predominantly enriched subcategories were “ion transport” (GO:0006811, part of BP), “extracellular region” (GO:0005576, part of CC), and “structural constituent of cuticle” (GO:0042302, part of MF). Among the up-regulated genes, the predominant enriched subcategory was “extracellular region”, which accounted for 34.42% of the total number of up-regulated genes. For down-regulated genes, “extracellular exosome” (11.07%) (FDR < 0.001) was predominantly enriched. These results suggest that Cd may induce damage to cellular components and their related functions in nervous tissue of spiders.

**Fig. 5:**
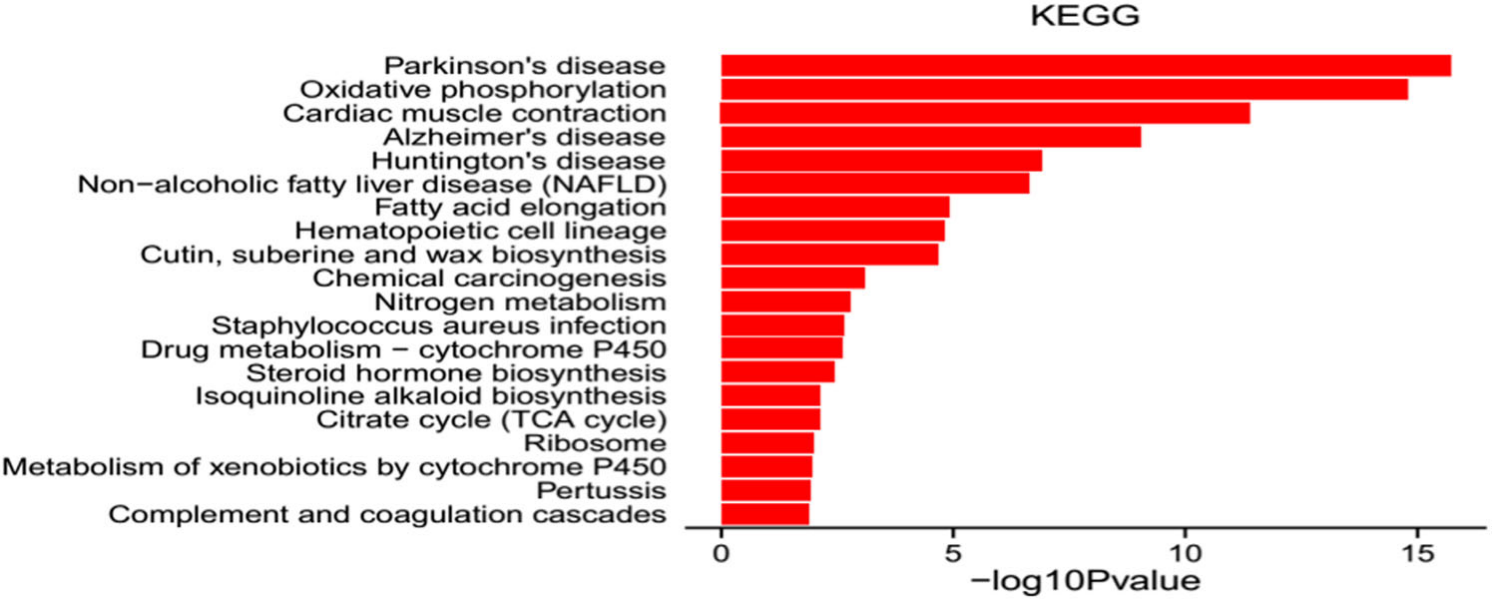
Enriched KEGG pathways that are represented in the DEGs of Cd-treated and control *P. pseudoannulata*

The KEGG annotation of DEGs identified 271 differently pathways, where 39 significantly enriched pathways were obtained. Under Cd stress, signaling transduction, energy supply processing, protein processing, and nervous diseases were significantly enriched (Fig. 5, Table 4). The results showed that the pathway with the lowest Q value was Parkinson’s disease, followed by Oxidative phosphorylation. Up-regulated pathways with maximum gene number were Calcium signaling pathway and cGMP—PKG signaling pathway, while down-regulated pathways were Oxidative phosphorylation and Parkinson’s disease (Table 4).

**Table 4:** The dominant and significantly enriched KEGG pathways in the DEGs of Cd-treated and control *P. pseudoannulata*

Ko ID	Term	Gene number	Enrichment score	Regulated
ko04020	Calcium signaling pathway	5	5.35	Up
ko04022	cGMP—PKG signaling pathway	5	4.50	Up
ko00350	Tyrosine metabolism	4	12.43	Up
ko04745	Phototransduction—fly	4	9.51	Up
ko04916	Melanogenesis	4	6.80	Up
ko00950	Isoquinoline alkaloid biosynthesis	4	22.19	Up
ko00190	Oxidative phosphorylation	41	4.53	Down
ko05012	Parkinson’s disease	41	4.19	Down
ko05010	Alzheimer’s disease	34	3.00	Down
ko05016	Huntington’s disease	34	2.41	Down
ko04932	Non-alcoholic fatty liver disease (NAFLD)	27	3.18	Down
ko03010	Ribosome	24	1.66	Down

### qPCR validation of transcriptome analysis

**Fig. 6:**
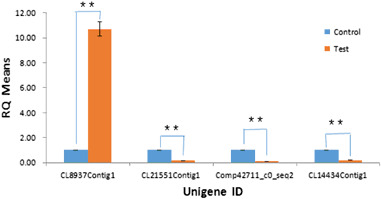
Validation of the RNA-Seq expression profiles of selected DEGs by qPCR

In order to verify the accuracy of the differential expression analysis of the transcriptome, four up-regulated or down-regulated genes were randomly selected for qPCR analysis. The qPCR results revealed that in the test group the expression of Comp208328_c0_seq4Contig1 transcript was 10.7 times higher than that in the control group, while the down-regulated genes Comp91669_c0_seq1, Comp42711_c0_seq2 and Comp215590_c0_seq2 Contig1 transcripts were only 0.16, 0.11 and 0.2 fold of that in the control group, respectively (Fig. 6). The results are consistent with those observed in the transcriptome analysis, demonstrating the repeatability of our transcriptome data.

## Discussion

Previous whole-body transcriptome studies of *P. pseudoannulata* identified multiple candidate genes involved in the spider’s response to Cd stress, and also identified that Cd stress causes oxidative damage to digestive tissues and disrupts metabolic activities and signal transduction. Signaling pathways that were affected included Notch, MAPK, AMPK, Hedgehog, Ras and TNF. Cd stress also changed the expression of ABC transporters, and induced the expression of glutathione metabolism related enzymes, Hsp70 and Hsp20 in the spider (Li et al. 2016). The cerebral ganglion is involved in crucial physiological functions, such as foraging behavior, integrating sensory information, enabling the spider to respond appropriately to environmental stimuli, and ability of the spider to orient itself. In addition, it is well established that the activity of neurosecretory cells in the cerebral ganglion determines molting and sexual maturation (Foelix 2011). To better understand how Cd affects animals and their interactions with the environment, we sought to characterize the genes affected by Cd toxicity in the spider’s cerebral ganglion at the transcriptome level.

### Metabolism

Oxidative phosphorylation oxidizes nutrients to release ATP, which serve as a source of energy in the cell, and this process primarily occurs in the mitochondria of eukaryotic cells (Rédei 2008). We found that enzymes involved in oxidative phosphorylation processes experienced change in gene expression levels when spiders were treated with Cd. Enzymes included NADH dehydrogenase (ubiquinone), succinate dehydrogenase (ubiquinone) flavoprotein, ubiquinol-cytochrome c reductase, cytochrome c oxidase cbb3-type and F-type H+-transporting ATPase, which all showed lower expression levels under Cd stress. Of the OXPHOS-related genes that showed changes in expression after Cd-stress, 9/16 genes encoded NADH dehydrogenase (ubiquinone) and were expressed at lower levels. NADH dehydrogenase (ubiquinone), which is alternatively referred to as Complex I (EC 1.6.5.3) that catalyzes the transfer of electrons from NADH to coenzyme Q10 (CoQ10) (Nakamaru-Ogiso et al. 2010). Brandt (2006) indicated that Complex I is the largest and most complicated enzyme of the electron transport chain, which is located in the inner mitochondrial membrane. The detoxification processes of Cd are likely energy-intensive and affect the spider’s development, reproduction, biochemical and physiological processes (Li et al. 2016; Eraly et al. 2011; Wilczek et al. 2013). Cd may cause damage of nerve cells by reducing the levels of oxidative phosphorylation enzymes in the spider cerebral ganglion, leading to a shortage of energy there. Neurons have an overall high metabolic rate, therefore neurons may suffer more from heavy metal damage (Nava-Ruíz and Méndez-Armenta 2013). Sun et al. (2016) found changes in gene expression levels of oxidative phosphorylation enzymes in fresh water crabs when exposed to Cd. Another study also showed that oxidative phosphorylation was critical for spider’s to respond to high temperature stress (Xiao et al. 2016). The KEGG annotation of the DEGs identified pathway types for Parkinson’s disease (ko05012), Alzheimer’s disease (ko05010), and Huntington’s disease (ko05016) that were differentially expresed, indicating that Cd influenced changes in gene expression for genes that are involved in neurological diseases.

The primary effect of Cd exposure in animals appears to be oxidative stress caused by the accumulation of reactive oxygen species (ROS) (Itziou et al. 2011). Superoxide is a ROS that can lead to oxidative stress in the cell, and its accumulation is also linked to neuromuscular diseases and aging (Esterházy et al. 2008). These results were corroborated by previous studies that showed that Cd induces oxidative stress and mitochondrial dysfunction, leading to neurodegenerative disorders and nervous function disorders, such as Alzheimer’s disease and amyotrophic lateral sclerosis (Jiang et al. 2007; Bar-Sela et al. 2001). NADH dehydrogenase produces superoxide by transferring one electron from FMNH2 to oxygen (O_2_). Since the gene showed strong changes in expression under Cd stress, Cd-induced disruption of the cerebral ganglion of spider may result in disrupting superoxide production.

While most DEGs detected in this study were down-regulated, the tyrosine metabolism pathway encoding tyrosinase and tyrosine 3-monooxygenase showed up-regulation under Cd stress. Tyrosinase is involved in wide ranging processes in insects including wound healing and sclerotization, melanin synthesis and parasite encapsulation. Some commonly used insecticides inhibit tyrosinase, implying its importance in insect defensive mechanisms to stress (Kim and Uyama 2005). Nagatsu (1995) suggested that tyrosine 3-monooxygenase was a precursor for neurotransmitters norepinephrine (noradrenaline) and epinephrine (adrenaline), which were produced in the cerebral ganglion, peripheral sympathetic neurons and the adrenal medulla. Given that tyrosine metabolism pathway genes showed increased gene expression under Cd-treated conditions, these genes may play a role in defending spiders against Cd-induced damage to the nervous system.

### Signal transduction

Signal transduction is the process by which a chemical or physical signal is transmitted through a cell as a series of molecular events, and it is the basic mechanism controlling cell growth, proliferation, metabolism and many other processes (Jiang et al. 2007). Signal transduction plays important roles in heavy metal stress, including metal transport, metal regulation, and detoxification functions. (Leonard et al. 2004; Adams et al. 2002; Tian et al. 2015). Cd can interact with different hormonal signaling pathways, such as estrogen and MAPK signaling that can control proliferation, differentiation, and survival/death (Ali et al. 2010, 2012; Clapham 2007). Cd-stress primarily resulted in changes in gene expression of the calcium signaling pathway and the cGMP—PKG signaling pathway (Table 4). As a ubiquitous second messenger, calcium (Ca^2+^) plays a significant role in cellular signaling, and is particularly involved in neuronal functions such as muscle contractions, neuronal transmission, neurogenesis, and synaptic plasticity (Demaurex and Nunes 2016; Rash et al. 2016; Berridge et al. 2000). Ca^2+^ signaling is involved in response to metal stress in both plants and animals (Wang et al. 2011; Wang et al. 2014). Sun (2011) suggested that Ca^2+^ plays a crucial role in the apoptosis of rat cortical neurons induced by Cd exposure. KEGG analysis of DEGs identified the up-regulation of phosphatidylinositol phospholipase (PLCB), Ca^2+^ transporting ATPase (ATP2A) and solute carrier family 25 (ANT), and GO annotation of the enriched DEGs identified 14 GO terms including 20 genes related to “calcium signal”. These results suggest that the calcium signaling pathway could be actived or become regulated downstream of another pathway to contribute to the response of spider cerebral ganglia under metal stress. This result was consistent with the calcium signaling pathway quickly and effectively regulating downstream signaling and gene expression in response to low temperature stress in *P. pseudoannulata* (Xiao et al. 2016).

As noted above, one of the pathways that were significantly up-regulated under Cd stress was the the cGMP—PKG signaling pathway. Specifically, phosphoinositide phospholipase C (PLC) (EC 3.1.4.11) and Ca^2+^ transporting ATPase (PMCA) (EC:3.6.3.8) were significantly up-regulated in the cerebral ganglion of spiders when exposed to Cd. PLC belongs to a larger superfamily of phospholipases, which is a family of eukaryotic intracellular enzymes that plays an important role in signal transduction processes (Meldrum et al. 1991). PLC is an enzyme that facilitates the release of Ca^2+^, leading to increased intracellular Ca^2+^ levels, and facilitating cellular responses through stimulation of Ca^2+^-sensitive proteins such as Calmodulin. PMCA is a Ca^2+^ transport ATPase that localizes to the plasma membrane and facilitates the removal of Ca^2+^ from the cell, playing a viral role in regulating Ca^2+^ levels within cells (Jensen et al. 2004). PMCA is placed within three KEGG pathways that were significantly enriched, including calcium signaling pathway, cGMP—PKG signaling pathway, both of which were up-regulated, and Alzheimer’s disease, which was down-regulated. These results suggest that PMCA may be a key protein involved in response to Cd exposure in the cerebral ganglion of spiders. However, the underlying mechanism of metalloregulatory and detoxification in cerebral ganglion of spider under Cd stress at the protein level needs to be further studied.

In our analysis, we found up-regulation of genes encoding phototransduction-fly signaling related proteins, suggesting that Cd may interfere with the optical signal transduction in the spider. This is consistent with results of previous studies in which spiders exposed to Cd exhibited increased apoptosis in photoreceptors and ganglionic cells that led to phenotypic changes (Roozbehi et al. 2007).

### Genetic information processing

From the KEGG analysis of DEGs, 24 ribosome proteins were down-regulated in the cerebral ganglion of spiders exposed to Cd. The spider cerebral gaglion releases acetylcholine, approximately 10 different amino acids and several biogenic amines as neurotransmitters (Schmid et al. 1992). The ribosomal proteins levels decrease in spider exposed to environmental stress, such as high temperatures (Xiao et al. 2016), indicating which contributed to protein biosynthesis compensates upon stress. The down-regulation of ribosomal proteins may lead to damage in the spider cerebral ganglion by disrupting the synthesis of proteins related to neurotransmitter regulation.

## Conclusion

In summary, we generated transcriptomes of the cerebral ganglion of *P. pseudoannulata* under standard lab conditions, and under Cd stress. A total of 123,328 assembled unigenes were obtained, with an average length of 1040.73 bp. We identified 1441 Cd stress-associated DEGs in the cerebral ganglion of *P. pseudoannulata*, and 39 different significantly enriched pathways involving metabolic processes, signaling transduction, protein processing, and nervous diseases. We found that exposure to Cd leads to changes in the expression of genes involved in oxidative phosphorylation-associated, calcium signaling-associated, cGMP-PKG signaling, and nervous disease-associated and ribosomal-associated genes. Overall, the our results facilitated the discovery of genes that respond to the neurotoxic effects of Cd and in the identification of biomarkers for monitoring heavy metal pollution.

## Electronic supplementary material

Supplementary Information
