# Supplementary material for: Transcriptome assembly and expression profiling of the molecular responses to cadmium toxicity in cerebral ganglia of wolf spider Pardosa pseudoannulata (Araneae: Lycosidae)
Source: Ecotoxicology. 2018 Jan 3;27(2):198–208. doi: 10.1007/s10646-017-1885-1 (PMC5847060; doi:10.1007/s10646-017-1885-1)
Supplement: Supplementary file 1 — Supplementary Information [file 10646_2017_1885_MOESM1_ESM.doc]

**Supporting Information**


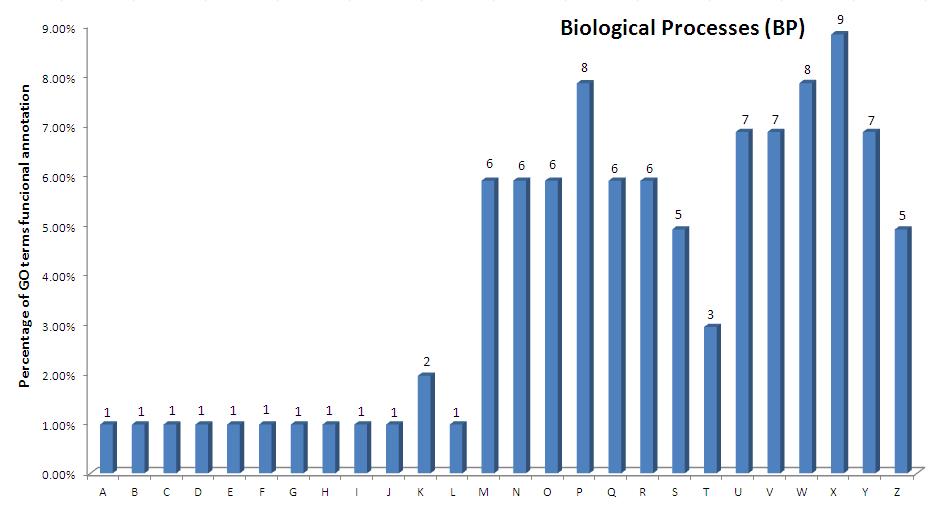


A: skeletal muscle thin filament assembly

B: positive regulation of superoxide anion generation

C: regulation of cell-cell adhesion mediated by integrin

D: mitochondrial ATP synthesis coupled proton transport

E: positive regulation by host of symbiont catalytic activity

F: positive regulation of sister chromatid cohesion

G: cellular response to ATP

H: cap snatching

I: response to antipsychotic drug

J: regulation of relaxation of cardiac muscle

K: positive regulation of oxidative stress-induced intrinsic apoptotic signaling pathway

L: regulation of multicellular organismal development

M: viral RNA genome replication

N: extracellular fibril organization

O: elastic fiber assembly

P: cellular iron ion homeostasis

Q: collagen metabolic process

R: regulation of JUN kinase activity

S: fusion of sperm to egg plasma membrane

T: fatty acid elongation, monounsaturated fatty acid

U: chitin metabolic process

V: fatty acid elongation, saturated fatty acid

W: phototransduction

X: proteolysis involved in cellular protein catabolic process

Y: very long-chain fatty acid biosynthetic process

Z: paranodal junction assembly

Fig. S1 BP categories of significantly enriched GO terms assigned to DEGs in the cerebral ganglion transcriptome of Cd- and non-Cd treated *P. pseudoannulata*. Numbers on the bar indicate the sum of annotated DEGs.


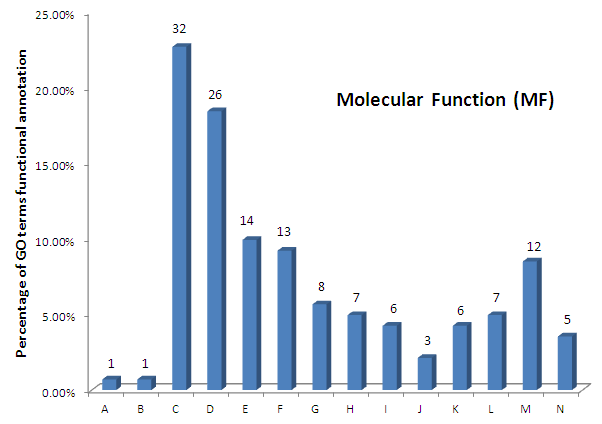


A: L-lactate dehydrogenase activity

B: vitamin D binding

C: structural constituent of cuticle

D: chitin binding

E: cytochrome-c oxidase activity

F: NADH dehydrogenase (ubiquinone) activity

G: RNA-directed RNA polymerase activity

H: photoreceptor activity

I: peptidase inhibitor activity

J: fatty acid elongase activity

K: fatty-acyl-CoA reductase (alcohol-forming) activity

L: cysteine-type endopeptidase inhibitor activity

M: transferase activity

N: long-chain-fatty-acyl-CoA reductase activity

Fig. S2 MF categories of significantly enriched GO terms assigned to DEGs in the cerebral ganglion transcriptome of Cd- and non-Cd treated *P. pseudoannulata*. Numbers on the bar indicate the sum of annotated DEGs.


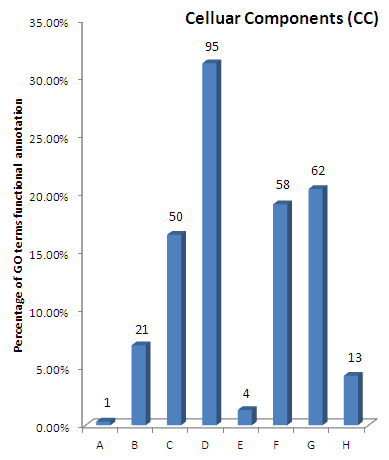


A: sperm fibrous sheath

B: respiratory chain

C: mitochondrial inner membrane

D: extracellular region

E: other organism presynaptic membrane

F: extracellular space

G: endoplasmic reticulum membrane

H: myelin sheath

Fig. S3 CC categories of significantly enriched GO terms assigned to DEGs in the cerebral ganglion transcriptome of Cd- and non-Cd treated *P. pseudoannulata*. Numbers on the bar indicate the sum of annotated DEGs.
